# Systemic Actions of SGLT2 Inhibition on Chronic mTOR Activation as a Shared Pathogenic Mechanism between Alzheimer’s Disease and Diabetes

**DOI:** 10.3390/biomedicines9050576

**Published:** 2021-05-19

**Authors:** Gabriela Dumitrita Stanciu, Razvan Nicolae Rusu, Veronica Bild, Leontina Elena Filipiuc, Bogdan-Ionel Tamba, Daniela Carmen Ababei

**Affiliations:** 1 Center for Advanced Research and Development in Experimental Medicine (CEMEX), Grigore T. Popa University of Medicine and Pharmacy, 16 Universitatii Street, 700115 Iasi, Romania; gabriela-dumitrita.s@umfiasi.ro (G.D.S.); veronica.bild@gmail.com (V.B.); leontina.filipiuc@umfiasi.ro (L.E.F.); 2 Pharmacodynamics and Clinical Pharmacy Department, Grigore T. Popa University of Medicine and Pharmacy, 16 Universitatii Street, 700115 Iasi, Romania; razvan.nicolae.rusu@gmail.com (R.N.R.); dana.ababei@gmail.com (D.C.A.); 3 Department of Pharmacology, Clinical Pharmacology and Algesiology, Grigore T. Popa University of Medicine and Pharmacy, 16 Universitatii Street, 700115 Iasi, Romania

**Keywords:** Alzheimer’s disease, sodium glucose cotransporter 2 inhibition, mechanistic target of rapamycin, metabolic dysfunction hypothesis, diabetes

## Abstract

Alzheimer’s disease (AD) affects tens of millions of people worldwide. Despite the advances in understanding the disease, there is an increased urgency for pharmacological approaches able of impacting its onset and progression. With a multifactorial nature, high incidence and prevalence in later years of life, there is growing evidence highlighting a relationship between metabolic dysfunction related to diabetes and subject’s susceptibility to develop AD. The link seems so solid that sometimes AD and type 3 diabetes are used interchangeably. A candidate for a shared pathogenic mechanism linking these conditions is chronically-activated mechanistic target of rapamycin (mTOR). Chronic activation of unrestrained mTOR could be responsible for sustaining metabolic dysfunction that causes the breakdown of the blood-brain barrier, tau hyperphosphorylation and senile plaques formation in AD. It has been suggested that inhibition of sodium glucose cotransporter 2 (SGLT2) mediated by constant glucose loss, may restore mTOR cycle via nutrient-driven, preventing or even decreasing the AD progression. Currently, there is an unmet need for further research insight into molecular mechanisms that drive the onset and AD advancement as well as an increase in efforts to expand the testing of potential therapeutic strategies aimed to counteract disease progression in order to structure effective therapies.

## 1. Introduction

Alzheimer’s disease (AD), the leading cause of dementia in aging people, is characterized by a cognitive decline that involves memory, orientation, judgment, communication and reasoning, and is a major threat to people’s health and quality of life worldwide [1,2]. According to the World Health Organization, over 47 million people are afflicted by AD globally, and this number is expected to reach almost 76 million by 2030 and about 115 million by 2050 [3]. The incidence of AD continues to rise steadily; as aging demographics of the global human population and life expectancy are increasing, leading to a heavy economic and societal burden. Undoubtedly, extensive research into the pathogenesis and AD therapies continues to stimulate in-depth efforts by academia, pharmaceutical companies and government attention to finding curative compounds or at least slow the disease progression.

**Figure 1:**
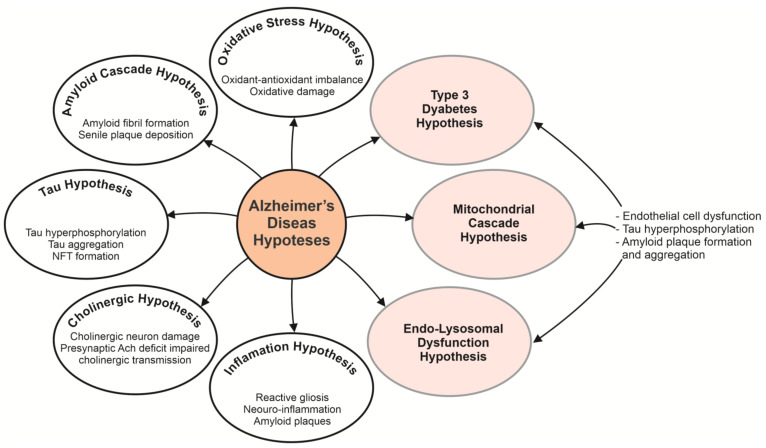
Alzheimer’s disease is a neurodegenerative disease that involves a multitude of factors. Given the complexity of the human brain, the lack of effective research tools and reasonable animal models, the detailed pathophysiology of the disease remains unclear. Based on multifaceted nature of AD, there have been proposed various hypotheses, including Aβ aggregation, cholinergic dysfunction, tau aggregation, oxidative stress, inflammation, etc. Challenges and future prospects include extensive testing of new hypotheses such as endo-lysosomal, mitochondrial and metabolic dysfunctions to attack the disease from different angles for the effective development of an early diagnosis and successful drugs for therapies. NTF, neurofibrillary tangle; Ach, acetylcholine.

To date, with a multifactorial nature, the ultimate cause of AD remains elusive, and is generally considered to be related to genetic, neuroendocrine, biochemical, environmental, and immune factors based on aging [4,5]. In recent decades, several hypotheses have been designed to explain the AD pathogenesis mechanisms, such as amyloid-β (Aβ) deposition as the core of neuritic plaques, tau protein hyperphosphorylation as the key constituent of neurofibrillary tangles, degeneration of cholinergic neurons, or death [2,6,7,8,9]. Figure 1 highlights some of the most studied hypotheses for AD, including Aβ aggregation [10,11,12], cholinergic dysfunction [13,14], tau aggregation [15,16], oxidative stress [17,18], inflammation [19,20,21]. Challenges and future prospects include extensive testing of new hypotheses such as endo-lysosomal [22,23,24], mitochondrial [25,26,27,28] and metabolic dysfunctions [29,30]. It remains to be determined whether the root cause of AD is the Aβ aggregates formation and accretion between neurons or tau neurofibrillary tangle developments within neurons or the cumulative end-effects of other causal epigenetic and/or genetic processes, or a fusion of both [10,31,32,33]. In addition, growing evidence suggests that endo-lysosomal, mitochondrial and metabolic dysfunction display a critical role in the multiple memory and attention processes of the elderly and are viable early drivers in the onset and progression of AD [34,35,36]. Thus, it is more and more evident that there is a solid interplay between metabolic dysfunction related to metabolic syndrome, diabetes, obesity and patient’s susceptibility to AD development [37,38]. From the strong relationship between AD and the pathological conditions of diabetes mellitus, AD can be referred to as “diabetes type 3” or “brain diabetes” [39,40].

Overwhelming results suggest that there are early abnormalities in cerebral glucose metabolism in people with AD [41,42], involving deficiencies in glycolysis and glucose transporters [43,44]. A candidate for a shared pathogenic mechanism linking these metabolically-driven conditions is represented by a chronic mechanistic target of rapamycin (mTOR) signaling activation [45]. Chronic unrestrained mTOR activation may be behind AD lysosomal, mitochondrial and metabolic alteration, causing the failure of the blood-brain barrier (BBB) through endothelial cell dysfunction; as well as leading to tau hyperphosphorylation, amyloid plaques formation and aggregation in the brain [38,46]. Inhibition of sodium glucose cotransporter 2 (SGLT2), facilitated by a constant glucose loss, is thought to restore the mTOR cycle via nutrient-driven, nocturnal periods of transient mTOR inhibition (catabolism) interferes by transient mTOR activation (anabolism) during daily accompanying nutrition. Thus, a flexible dynamic of mTOR is reinstated, preventing or arresting AD progression. The current paper aims to discuss the possible implications of SGLT2 inhibition on chronic activation of mTOR as a common pathogenic mechanism between AD and diabetes, according to the recent research findings.

## 2. Pharmacological Approaches Able of Impacting Alzheimer’s Disease and Its Progression

Given the poor epidemiological forecast and the increasing number of experimental and clinical evidence that send the manifestations of AD beyond the brain, there is a major research interest in expanding testing of new hypotheses to attack the disease from different angles to provide insights into novel therapeutic strategies. Three such hypotheses are diabetes type 3, the mitochondrial cascade hypothesis and the endo-lysosomal dysfunction hypothesis. They provide a basis for therapeutic approaches to restore AD-related metabolic, mitochondrial and endo-lysosomal dysfunctions; changes that occur early in the progression of the disease in relation to tau and Aβ deposition, which means that they are viable factors of AD development [34,35].

In AD patients, factors related to mitochondrial functions have been severely compromised. Such factors include mitochondrial morphology, oxidative phosphorylation, Ca^2+^ buffering, mitochondrial biogenesis and transport along the neuronal axon. These processes could lead to negative consequences for neurons as well as for the whole structure of the brain. Mitochondria are organelles that are defined as the powerhouse of the cell due to the fact that cells in the human body rely on them to provide energy for vital functions. Neurons depend on the presence of mitochondria especially at the synapses where these organelles produce adenosine triphosphate (ATP) while also buffering calcium (Ca^2+^) ion concentration. Thus, the high number of mitochondria located in the synaptic area is justified, compared to other parts of neurons [27,47]. The activity of enzymes involved in mitochondrial energy production is decreased in AD brains, thus contributing to the compromise of the mitochondrial ATP production. In line with this observation is the fact that mild cognitive impairment which is one of the early stages in AD chronology is associated with an increased level of oxidative stress markers and a decreased level of antioxidants in the brain and peripheral compartments [27,48]. This suggests there is a strong connection between oxidative stress and mitochondrial dysfunction. The oxidation of ATP synthase, a mitochondrial enzyme involved in oxidative phosphorylation has been found in isolated lymphocytes from AD peripheral blood as well as in AD brains, thus explaining the compromised activity of the ATP synthase and the reduction of ATP levels in AD. Another mitochondrial factor that is modified in AD is related to the dynamics of the mitochondria and processes such as fusion and fission. The unbalance of these processes led to compromised morphology and distribution of the mitochondria in neurons, fragmented mitochondria being observed in fibroblasts and brains from AD patients [27]. Due to their involvement in production of reactive oxygen species (ROS), mitochondria developed a system that can cope with damage done by ROS to its contents. The degradation at the organelle level is realized through a process called mitophagy. Studies have shown that inadequate mitophagy activity in eliminating increased number of damaged mitochondria led to disturbance in mitochondrial homeostasis, thus showing the involvement of the mitophagy process in AD [26].

The endo-lysosomal dysfunction hypothesis refers to the endo-lysosomal and autophagy system which is involved in maintaining protein homeostasis in cells. This system consists of endosomes, retromers, autophagosomes and lysosomes, each with its specific set of functions. One of AD’s vulnerable brain regions is the hippocampus. It is here that different factors related to the endo-lysosomal and autophagy system have been reported: increased number of endosomal compartments, abnormal accumulation of autophagic vacuoles and altered expression levels of protein degradation key regulators [49]. Abnormal functions of the endo-lysosomal and autophagic networks are common in AD due to their implication in the homeostasis of Aβ and tau [50]. Lysosomes are involved in degrading and recycling macromolecules, thus leading to generation of nutrients. They are the last step to degrading organelles, macromolecules or protein aggregates by the endocytic and autophagic pathway [51,52]. Being one of the main mechanisms of cellular waste removal, it is expected that genes that facilitate lysosomal degradation are linked to a broad number of diseases and factors such as enzymatic dysfunction and positioning regarding lysosomes are involved in neurodegenerative disorders. A common histopathological feature of AD is swollen, dystrophic neurites, with lysosomes accumulated within the axonal swellings, these swellings being located in regions proximal to Aβ plaques in patient brains [51,53,54]. Studies suggest that amyloid accumulation can be actively determined by abnormal lysosome axonal transport. Impaired lysosomal positioning may be a contributing factor in AD, this being confirmed by evidence of accumulation of axonal lysosomes, increased amyloid plaque burden and lysosome dysfunction [51]. Disruption of the endo-lysosomal system is one of the earliest detectable histopathological features of AD, abnormal transport and positioning of lysosomes being contributors to the pathogenesis of the disease [55].

The third hypothesis refers to AD as “diabetes type 3” due to the implications of insulin resistance within the brain and its impact on neuro-cognition, thus contributing to neurodegenerative diseases [56,57]. It seems that the metabolic dysfunction that characterizes obesity, type 2 diabetes mellitus and metabolic syndrome determines susceptibility for individuals to develop AD [58,59]. To understand the relationship between diabetes and neurodegenerative diseases it is important to know the role of energy homeostasis in diabetes. Differentiated neurons do not have the ability to regenerate. Lack of ATP moieties, energy crisis or oxidative stress will lead to their death or degeneration, causing neurodegenerative diseases [60]. Another important aspect is that more than 40% of ATP is used to maintain neurons viable or alive. Impaired glucose uptake is a result of compromised glucose metabolism in the brain, this eventually leading to glucose homeostasis alteration, which is an important factor in the pathogenesis of AD. Reduced levels of insulin in the central nervous system can determine overproduction and impaired clearance of Aβ and reduced levels of anti-amylogenic proteins [56]. Brain insulin resistance is the failure of brain cells to respond to insulin, this leading to insulin deficiency and impaired glucose transport inside the neurons. Insulin resistance in the central nervous system correlates with peripheral insulin resistance. Therefore, without the protective effect of insulin, neurons could be more susceptible to neurotoxic insults [61,62]. Insulin resistance in AD and diabetes can lead to hyperinsulinemia. Therefore, the insulin-degrading enzymes (IDE) can be saturated which can lead to defects in regulating levels of insulin, Aβ protein and amyloid precursor protein (APP), IDE being involved in the regulation of Aβ protein and APP levels [56,63]. In addition to its peripheral actions, insulin is involved in other processes such as inducing dendritic sprouting, cell growth and repair, neuronal stem cell activation. It appears that the neuroprotective effects of insulin are due to the regulation of phosphorylated tau levels. An increased level of insulin resistance is also associated with high levels of proinflammatory cytokines which are linked to Aβ depositions in the brains [64]. In diabetes, insulin resistance causes mitochondrial dysfunction, triggering inflammatory response with increased levels of cytokines such as interleukin (Il)-1β, Il-6, Il-8, tumor necrosis factor-alpha (TNF-α), alpha-1-antichymotrypsin (ACT) and C-reactive protein (CRP), the same mechanism being triggered in AD [65]. The common situation for both type 1 and 2 diabetes is chronic hyperglycemia, considered a risk factor for AD. Regarding AD, type 1 diabetes insulin deficiency seems to be the main factor for increased tau phosphorylation, while hyperglycemia-induced tau cleavage with insulin disturbances could be the factor that leads to tau pathology in type 2 diabetes [66]. The underlying mechanism that links these three hypotheses may be chronically-activated mTOR signaling, which influences mitochondrial dynamics, biogenesis and processes such as autophagy, mitophagy and proteostasis [47]. This activation is associated with physical inactivity and over-nutrition, which leads to chronic anabolic signaling driven by increased levels of glucose, amino acids and growth signaling factors prevalent in patients with metabolic conditions. By caloric restriction, increased activity, intermittent fasting or pharmacological agents capable of mimicking the interventions above-mentioned, the beneficial influence it would have on mTOR could lead to a positive impact on the progression of AD [58,67].

## 3. Implications of Restoring Metabolic Health in the Therapy of Alzheimer’s Disease

Energy production in the brain depends largely on glucose metabolism, as the disruption of its homeostasis would endanger neuronal cells. Both hyperglycemia and hypoglycemia affect the integrity of the brain, especially the cognitive functions. Cerebral glucose metabolism consists of glucose transport and intracellular oxidative catabolism, as the damage of this metabolism favors the appearance of metabolic abnormalities highlighted in the brain of patients suffering from AD. In this regard, it appears that glucose transport abnormalities may be related to insulin resistance [68], the defects in glucose transporters and glycogenolysis [43].

Metabolic dysfunction is a well-recognized risk factor for dementia, and particularly patients with diabetes seem to have an increased risk of AD [69]. This risk may be due to a shared pathogenic mechanism between AD and diabetes involving hyperinsulinemia and hyperglycemia, which raises the question of whether the use of antidiabetic compounds could impact the risk of dementia, and whether these agents may be used to prevent or treat AD [70]. Acetylcholinesterase (AChE) inhibitors, as primary targets for Alzheimer’s therapy, still offer symptomatic relief only, with no slowing of AD progression [71]. Thus, some recent studies explored the extent to which antidiabetic treatments could influence brain pathology, mainly AD characteristics (Table 1), with a majority of them targeting possible benefits on neuroinflammation, amyloid pathology, tau pathology, cognitive function, neurogenesis, oxidative stress or synapses [72,73,74,75]. A current nested case control research evaluating the implications of a range of antidiabetic drugs in dementia has shown that sulphonylureas/glinides, insulin, and thiazolidinediones (TZDs) had no positive impact on development of dementia. In contrast, dipeptidyl peptidase-4 (DPP-4) inhibitors, metformin, SGLT2 inhibitors and glucagon-like peptide-1 (GLP1) agonists showed benefit, with metformin barely reaching significance, whereas both SGLT2 inhibitors and GLP1 agonists use displayed a 42% decrease in dementia risk [76]. Metformin, a widely used biguanide, crosses the BBB and can improve various cognitive functions. An in vivo study of a diabetic mouse model treated with metformin found that it reduces hippocampal apoptosis, increases the expression of p- adenosine 5′mmonophosphate-activated protein kinase (AMPK), a protein involved in regulating energy metabolism, reduces vascular permeability, and stimulates endothelial nitric oxide synthesis [77]. Another study relates the neuromodulatory action of metformin, by activating various molecular signaling pathways with improved cognitive function such as memory in a streptozocin-induced diabetic rat model. After 8 weeks of treatment, the cognitive decline of diabetic rats was ameliorated and some of the therapeutic success would be due to the hypoglycemic effect of metformin [78].

Insulin resistance has also been associated with elevated levels of proinflammatory cytokines (Il-1, Il-6, TNF-α). Insulin signaling involves the brain to take up glucose and synthesize the insulin-degrading enzyme and is also involved in the degradation of β amyloid. In diabetes, due to the change in insulin signaling, a low synthesis of the enzyme involved in its degradation takes place, thus reducing the process of degradation of β-amyloid with abnormal accumulation in the brain [46,64,104,105]. Insulin and insulin-like growth factor (IGF) are hormones that regulate cell metabolism. These hormones in the brain are needed for the synaptic activity, neurogenesis, neuronal survival and memory. Synthesized in the pancreas, they cross the BBB and reach the brain, bind to insulin receptors and its growth factor followed by autophosphorylation under the action of kinases, affecting a number of cellular signaling pathways including PI3K/AKT, MAPK/ERK. S6, a downstream target of mTOR acts as negative feedback, phosphorylates and deactivates insulin growth factor substrates [46]. Recent studies have focused on the effects of insulin and its growth factor on β-amyloid accumulation. Some studies show that reduced signaling of insulin growth factor has a protective effect against the accumulation of beta amyloid while other studies have shown that in the brains of patients with postmortem AD, insulin resistance and reduced insulin signaling have been correlated with increased risk of dementia and AD [106,107]. Although the physiological role of insulin in the brain is incompletely understood, the intranasal insulin-based therapy began to attract attention in AD research, when small human studies described improved knowledge without a change in blood glucose or insulin levels in healthy volunteers [85,108]. Therapeutically, antidiabetic agents such as rosiglitazone and pioglitazone have been recommended, peroxisome proliferator-activated receptors (PPARy) agonists used to treat diabetes in order to improve the pathogenesis of insulin resistance and hyperglycemia [109]. PPARγ is a nuclear receptor with an essential role as a transcription factor in the control of inflammatory genes; PPARg agonists can inhibit these proinflammatory genes, as demonstrated in animal models of AD transgenic mice. These agonists reduced microglial inflammation and favored Aβ phagocytosis followed by improved cognitive function. The effectiveness of pioglitazone was demonstrated in a diabetic mouse model when the inflammatory responses present in AD were reduced. However, Phase III clinical trials for rosiglitazone and pioglitazone approved for the treatment of type 2 diabetes have failed due to lack of efficacy in AD, both of which have no impact on the disease [46,110]. Currently, type 2 diabetes therapy aims to reduce plasma glucose levels during the day by constantly discharging glucose into the urine and modifying sodium in the kidneys, SGLT2 inhibitors demonstrate a positive impact on anabolic/catabolic cycle restoration, a new way to treat AD [106]. SGLT2 inhibitors target the sodium-glucose cotransporter 2, the major glucose transporter in the kidney, responsible for the reabsorption of 90% of glucose from primary urine. Inhibition of SGLT2 decreases glucose reabsorption and thus increases urinary glucose excretion, leading to a reduction in both fasting and postprandial hyperglycemia; preventing glucotoxicity and hyperglycemia-induced damage [43]. The first clinical trial exploring the SGLT2 inhibition effects on AD patients is ongoing and focuses on brain energy metabolism impact following therapy with the SGLT2 inhibitor dapagliflozin [111]. Canagliflozin (known as Invokana) is SGLT2 targeting drug. A recent research discussed the canagliflozin effects on cerebral AChE activity in obese diabetic rats [103], while an enzoinformatics study was suggested as AChE inhibitor [112]. Recently, a new SGLT2i mechanistic theory was approached, which claims that the loss of glucose through urine directed by SGLT2 inhibitors restores the diurnal switching between anabolic and catabolic states caused by mTOR signaling [67].

mTOR is a serine/threonine (289 kDa) protein kinase with large dimensions present in all cell types, a protein named after rapamycin, a compound isolated in 1972 from *Streptomyces hygroscopicus* structurally related to lipid kinases such as phosphatidylinositol-3-OH kinase (PI3K), with a key role in multiple cellular processes such as glucose metabolism, apoptosis, proliferation, transcription and cell migration [113,114,115]. mTOR kinases function as a hub for switching between anabolic and catabolic processes, consisting of 2 complexes called mTORC1 and mTORC2, with different cellular functions and essential for life. mTOR binds to specific proteins in each complex (Raptor and Rictor), mTOR complex (mTORC)1 being activated by the availability of nutrients, especially amino acids and coordinates protein synthesis and degradation and mTORC2 being receptive especially to insulin, promoting stress responses, mediates conversation between pathways insulin signaling and mTOR signaling [116,117]. The target of mTOR is a protein kinase with an essential role in controlling protein synthesis, cellular functions and autophagic regulation, as the disorder of this major regulator is associated with the pathogenesis of various human diseases such as AD by Aβ deposition, deterioration of the metabolic state of the cell with the onset of diabetes and obesity, the inactivation of mTOR signaling being initiated in the early stages of AD [107].

Diabetes and AD are both linked to a condition of chronically activated mTOR, resulting in chronic inhibition of autophagic and lysosomal processes that affect the long-term functioning of the brain, pancreas, heart, kidney, and other organs [118,119,120]. Identifying which compound, if any, is ideal for the treatment of AD and whether these drugs would be optimal in association use, remains to be tested.

## 4. Impact of SGLT2 Inhibition on Chronic mTOR Activation: Is the Brain a Target?

**Figure 2:**
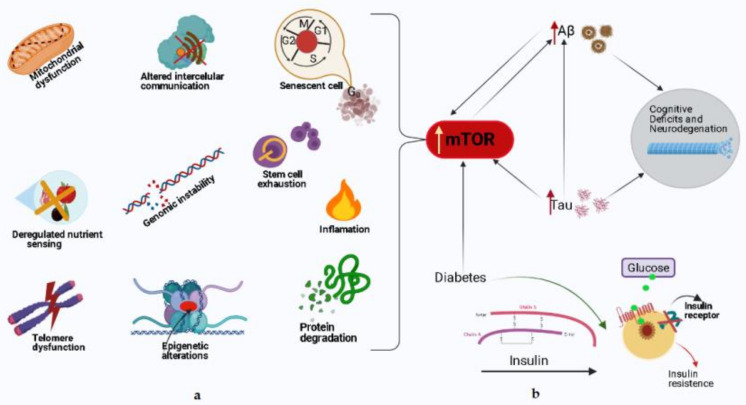
Schematic representation of mTOR hyperactivity in cognitive aging and AD. (**a**) Left—The implications of mTOR in main processes of aging. These features of aging, to different degrees, lead to an increased risk for AD, as well as cognitive decline during normal aging. Rapamycin and other pharmacological approaches that decrease mTOR activity may be valuable for delaying AD progression. (**b**) Right—The interrelation between neuropathological hallmarks of AD and mTOR. Hyperactive mTOR increases the production of Aβ and tau; and many factors including diabetes may influence the crosstalk of these proteins, and the aberrant cycle it creates contributes to the pathogenesis of AD.

mTOR activity is indispensable in terms of the normal cognitive process, while mTOR hyperactivity can be damaging to brain function [121,122,123]. The interrelation between neuropathological hallmarks of AD and mTOR has been studied extensively, highlighting a preclinical picture that often revealed contradictory-appearing data [124,125]. Figure 2 shows schematically the implications of mTOR hyperactivity in the normal cognitive process and AD.

Analyzing the changes of mTOR signaling in AD transgenic mouse models, Lafay-Chebassier et al. [126] reported lower mTOR signaling and an important alteration of mTOR phosphorylation in the cerebellum of 12-month-old APP/PS1 mice than controls, contradicting a previous study that revealed hyperactive mTOR signaling in 9-month-old APP/PS1 mice. The hyperactivity of mTOR has been described when the mice have extensive Aβ plaque deposits [127]. In a study that explored the correlation between the mTOR pathway and Aβ-induced synaptic dysfunction, which is considered to be critical in the AD pathogenesis; mTOR signaling was downregulated in young pre-pathological Tg2576 mice. In contrast, in elderly Tg2575 mice with established Aβ pathology, mTOR activity was comparable to that of wild-type mice of the same age [128]. Using 3xTg-AD mice, other studies have shown an age- and cerebral region-dependent increase in mTOR activity. The results showed that the formation of Aβ plaques preceded mTOR hyperactivity and was most likely due to high levels of soluble Aβ. Genetic or immunological prevention of Aβ formation and deposition was sufficient to decrease mTOR signaling to wild-type levels [129,130,131]. The findings were in agreement with reports exhibiting an upregulation in mTOR signaling in postmortem human brains affected by AD [132,133,134,135,136]. Chronic inhibition of mTOR by rapamycin therapy when it began in the early stage of Aβ deposition and in the absence of microtubule-associated protein tau (MAPT) pathology improved learning and memory function in transgenic mice modeling the disease [114,137].

Rapamycin administration both early and late in AD pathogenesis has been shown to delay, but not reverse accumulation of Aβ and MAPT tangles, as well as cognitive deficits in transgenic mouse models [122]. Although the data indicate that rapamycin treatment has unwanted side effects in the elderly population, therapies in which the compound is utilized in on-off programs may be designed for early or moderate AD stages. Additionally, research using agents other than rapamycin that inhibit the mTOR pathway and lack its side effects may be justified. While it is difficult to dissect the underlying causes of these divergent findings, the strain and age of animals, as well as variable Aβ levels may have differential effects on mTOR. Recent data suggests that, just as Aβ affects mTOR, mTOR similarly affects Aβ. This indicates that these proteins are closely correlated with each other and clarification of the mechanism of this relationship may reveal previously unknown features of AD pathogenesis [45].

**Figure 3:**
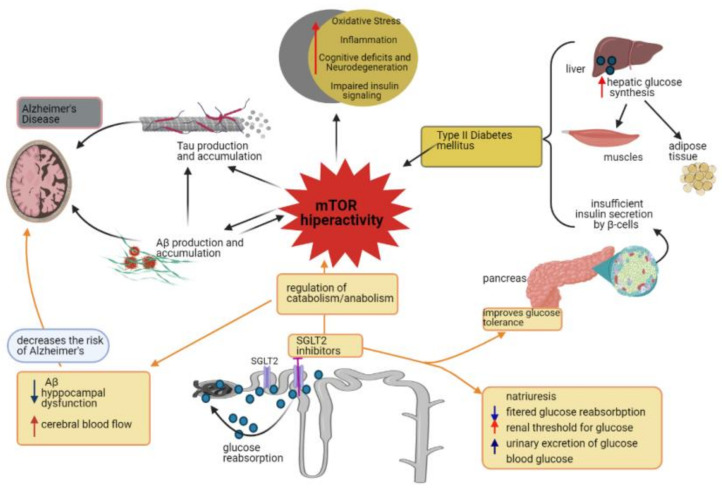
Type 2 diabetes is characterized by insulin resistance caused by uncontrolled hepatic glucose synthesis and by reduced uptake of glucose by muscle and adipose tissue. The pancreas contains functional β cells, but the variable secretion of insulin affects the maintenance of glucose homeostasis because β cells are gradually reduced. AD is characterized by increased synthesis and accumulation of tau and β-amyloid proteins. Aβ plaques may induce insulin resistance. Cerebral glucose metabolism consists of glucose transport and intracellular oxidative catabolism, affecting this metabolism favoring the appearance of metabolic abnormalities highlighted in the brains of patients with AD. Chronic activation of mTOR may be responsible for as endo-lysosomal, mitochondrial and metabolic dysfunctions in AD. High glucose intake causes hyperactivation of mTOR with abnormal insulin signaling accompanied by accelerated progression and symptoms similar to AD and with hyperglycemia and the appearance of type 2 diabetes. In patients with type 2 diabetes and AD it occurs: increased oxidative stress, inflammation, cognitive deficit and insulin resistance. Type 2 diabetes therapies based on type 2 co-transport inhibitors for sodium and glucose promotes: natriuresis, reduced filtered glucose reabsorption, decreased renal threshold for glucose, increased urinary glucose excretion followed by reduced plasma glucose levels. These compounds have a positive impact on the restoration of the anabolic/catabolic cycle and represent a new way to treat AD. AD, Alzheimer’s disease; Aβ, amyloid β; SGLT2, sodium glucose cotransporter 2; mTOR, mechanistic target of rapamycin.

Protein synthesis and their degradation controlled by the autophagy process, the mechanistic target of mTOR, is a main switch that integrates growth factors and the state of cellular nutrients that influence metabolism, modulate aging [133,138]. Reduced mTOR signaling may be a mechanism by which dietary restriction leads to increased longevity, compensating for reduced aging time [116,117,139]. Autophagy is a lysosome-dependent homeostatic process by which toxic compounds, damaged organelles and mitochondria, misfolded proteins are sequestered in autophagosomes, with vital roles in various physiological and pathological processes such as cell death and the elimination of pathogenic microorganisms or protein accumulation in cells followed by neurodegeneration [140,141,142]. The mTOR signaling pathway seems to be involved in both type 1 and type 2 diabetes, insulin production being reduced in type 1 diabetes due to the destruction of pancreatic β cells while insulin resistance occurs in type 2. The survival of β cells depends on the regulation of the insulin receptor substrate -2 (IRS), the chronic exposure of these cells to glucose and an increased phosphorylation of Ser/Thr being correlated with the decrease in the level of the IRS-2. Insulin-induced protein proliferation and glucose- and amino acid-induced growth are dependent on mTOR signaling in pancreatic cells, as chronic mTOR activation results in insulin resistance characterized by hyperglycemia, and the onset of type 2 diabetes [143] (Figure 3).

In therapy with SGLT2 inhibitors, the uric acid levels decrease early in conjunction with other inflammatory markers, such as high-sensitive CRP, suggesting an early influence on oxidative stress/inflammation-associated processes. Uric acid is recognized as a mediator of endothelial dysfunction and inflammation through its activation of the nod-like receptor pyrin domain containing 3 (NLRP3) inflammasome [144,145,146]. Activation of NLRP3 in the microglia is a key stress-induced innate immune mechanism that leads to AD pathology [147,148]. The detailed mechanism by which SGLT2 inhibitors decrease uric acid is currently unknown, but it is interesting that its elevated levels have been shown to indirectly activate mTOR [149]. Its rapid and persistent decrease caused by SGLT2 inhibitors, in patients with elevated uric acid levels, offers another possible mechanism to reduce chronically activated mTOR signaling. Even so, the role of uric acid in the development of neurodegenerative diseases is not clearly defined. Higher uric acid levels can positively influence cognitive function and reduce the risk of AD onset and progression [150,151].

A growing body of evidence suggests that reduced nitric oxide (NO) signaling is involved in AD-related pathological processes [152,153]. The NO production is diminished via endothelial (e) nitric oxide synthase (NOS) phosphorylation, resulting in uncoupling of NO production [154]. It has been shown that mTOR hyperactivity uncouples NO production through eNOS phosphorylation, thus increasing superoxide generation. With a key role in maintaining endothelial function, chronic disruption of NO production can lead to inflammation, oxidative stress and endothelial dysfunction [154,155]. Dietary rapamycin supplementation has been shown to reverse age-related vascular endothelial dysfunction and oxidative stress accompanied by a decrease in superoxide production similar to levels in younger animals [156]. These results suggest the potential for SGLT2-driven mTOR inhibition in endothelial cells at the BBB level to modulate the dysfunction and oxidative stress linked with chronic mTOR activation and to reinstate properly endothelial function and NO production.

The most essential amino acids that activate mTOR in order to prevent the formation of autophagosomes are leucine, glutamine and arginine. A decrease in the level of these amino acids also seems to drive the lysosomal acidification process critical for protein degradation independent of autophagy activation [157]. Remarkably, amino acid starvation appears to be a faster and stronger activator of lysosomal/autophagy degradation than rapamycin, a direct pharmacological inhibitor of mTOR, making SGLT2 inhibitors potentially superior options to rapamycin in treating disorders characterized by chronic mTOR activation [158]. Clinical data showing an increase in amino acid catabolism during use of SGLT2 inhibitors is suggested by the increased oxidation of proteins, which is evident following 3 months of dapagliflozin therapy [159]. The increase in urea and urea cycle metabolites evident in a study in diabetic patients treated for 30 days with empagliflozin also suggests that there is a growth in protein catabolism [160].

Moreover, recent data evaluating the post-mortem status of mTOR in the brain of the patient with AD revealed concurrent phosphorylation/activation of both AMPK and mTOR which were co-localized with hyperphosphorylated tau. The results of this study suggest that the concurrent dysregulated AMPK activity that causes chronic mTOR activation is critical for genesis and progression of AD, and fundamentally driven by a lack of constant periods of fasting amino acids flux to the liver to support gluconeogenesis [161]. The striking parallelism of these molecular, cellular, and clinical profiles occurring along the path towards AD could be beneficially impacted by restoration of circadian SGLT2 inhibition mTOR modulation.

## 5. Concluding Remarks

Precision therapies for AD, in which genetic, environmental, neuroendocrine, biochemical and immune data are included to design specific prevention and treatment strategies, lagged behind other areas such as neoplastic diseases. This gap is partly due to the fact that there is no strong consensus on which therapeutic approaches might be effective. With the emergence of new pharmaceutical options and the increasing availability of large sets of metabolic data, the targeted approaches are expected to become more feasible.

Activation/inhibition of mTOR activity may be a shared pathogenic link between all metabolic and mitochondrial dysfunctions in AD, influencing metabolic dynamics, mitochondrial activity and biogenesis (fusion/fission), and essential housekeeping processes (proteostatis, mitophagy and autophagy) facilitated via circadian nutrient flux.

These circadian anabolic and catabolic fluxes, specific to healthy people, are disturbed by aging, physical inactivity, over-nutrition and metabolic diseases leading to the idea that improvements in metabolic flow through either intermittent fasting, increased activity, caloric restriction or pharmacological compounds able of mimicking the physiology of intermittent fasting/exercise/caloric restriction on mTOR may play a critical role in AD progression.

The multifarious nature of metabolic/remodeling role in AD and related disorders will require further research. It is likely that various aspects of the restoration of circadian SGLT2-mTOR modulation, such as its effects on anabolic (cell growth, protein synthesis,) and catabolic (lysosomal function, autophagy) processes are responsible for sustaining metabolic dysfunction in AD. Restoring metabolic health is an attractive avenue to facilitate future therapies for the prevention and treatment of AD, as well as to promote the preservation of healthy brain and body aging throughout life.

## Figures and Tables

**Table 1:** Classes of antidiabetic compounds as potential therapies for Alzheimer’s disease.

Antidiabetic Drugs	Experimental Model	Findings	References
Insulin	rat model of intracerebroventricular streptozotocin (STZ) injection-induced cognitive dysfunction, intraventricular delivery of 0.5 units = 12 nmol of detemir	rescued STZ-induced cognitive decline	[79]
	patients with early AD or moderate cognitive impairment; intranasal delivery of 20 or 40 IU insulin	improved attention, verbal memory and functional status; modulation of Aβ peptide	[80,81,82,83]
	healthy volunteers, intranasal administration of 4 × 40 IU of insulin	improvement in memory and mood, increase regional cerebral blood flow in the putamen and the insular cortex	[84,85,86]
Metformin	neuronal cell lines under prolonged hyperinsulinemic conditions, various concentrations of metformin (0.4–3.2 mM)	insulin signaling resensitization, prevention of the molecular and pathological changes observed in AD neurons	[87]
	murine primary neurons (from tau transgenic mice and wild type), different concentration of metformin (2.5 mM or 10 nM)	reduction of tau phosphorylation	[88]
	transgenic mouse model of AD intraperitoneal delivery of 200 mg/kg metformin; or 350 mg/kg/day metformin delivered in drinking water for several months	amelioration of cognitive deficits, reduce Aβ plaque deposition attenuation of memory impairment	[73] [89]
	in older adults with an incident diagnosis of AD; 1–9, 10–29, 30–59, or ≥60 metformin prescriptions	more than 60 prescriptions were correlated with a slightly increased risk of developing AD	[72]
Thiazolidinediones	transgenic AD mouse model 0.03 mg/kg/day of leptin intranasal delivery + intraperitoneal administration of 10 mg/kg/day pioglitazone for 2 weeks	reduce brain Aβ levels and spatial memory impairments	[71]
	7 days gavage therapy with 40 mg/kg/day of pioglitazone	decrease glial inflammation and soluble Aβ1–42 peptide levels by 27%	[90]
	control trial in patients with AD and diabetes, doses of 15–30 mg pioglitazone for 6 months	cognitive deficits amelioration and stabilization of the disease in diabetics with AD	[91]
	pilot trial with AD patients without diabetes; daily 45 mg of pioglitazone	no important efficacy data were detected	[92]
	clinical trials; 2 to 8 mg of rosiglitazone, as adjunct therapy in AD patients	pro-cognitive effects	[93]
Glucagon-like peptide-1 receptor agonists	transgenic mouse model of AD intraperitoneal injection with 1 or 10 nmol/kg of lixisenatide for 10 weeks 10 nmol/kg lixisenatide for 60 days	prevented memory impairment, neuronal loss, and deterioration of synaptic plasticity reduction of amyloid plaques and neurofibrillary tangles	[94] [95]
	intraperitoneal injection with 2.5 or 25 nmol/kg of liraglutide for 10 weeks	reduce Aβ deposition by 40–50%, and decrease inflammatory response	[96]
	a pilot clinical trial in AD patients; daily subcutaneously injections of 0.6 mg liraglutide in the first week; hereafter 1.2 mg daily for another week before finally increasing to 1.8 mg daily (week 26)	brain glucose metabolism decline prevention; no important cognitive changes compared with placebo group	[97]
Dipeptidyl Peptidase-4 Inhibitors	transgenic mouse models of AD 20 mg/kg/day of sitagliptin for an 8-weeks period daily gavage of 5, 10 and 20 mg/kg sitagliptin for 12 weeks	pro-cognitive effects, reduction of Aβ deposits diminution of nitrosative stress and inflammation markers, reduction of Aβ deposition	[98] [99]
	daily oral administration of 5, 10, and 20 mg/kg linagliptin for 8 weeks	amelioration of cognitive deficits, diminution of Aβ42 levels, reduction of tau phosphorylation and neuroinflammation	[100]
	STZ-induced rat model of AD; 0.25, 0.5 and 1 mg/kg of saxagliptin in gavage delivery for 60 days	reduction of Aβ formation, a marked decrease of Aβ42 level and tau phosphorylation	[101]
	STZ- induced rat model of AD; daily orally doses of 2.5, 5 and 10 mg/kg vildagliptin for 30 days	attenuation of tau phosphorylation, Aβ and inflammatory markers	[102]
Sodium-glucose cotransporter 2 inhibitors	scopolamine-induced rat model of memory impairment; daily oral gavage of 10 mg/kg canagliflozin for 14 days	improvement of memory dysfunction	[103]

STZ, intracerebroventricular streptozotocin; AD, Alzheimer’s disease; Aβ, amyloid β.

## Data Availability

No new data were created or analyzed in this study. Data sharing is not applicable to this article.

## Abbreviations

AD	Alzheimer’s disease
Aβ	amyloid β
mTOR	mechanistic target of rapamycin kinase
SGLT2	sodium glucose cotransporter 2
APP/PS1 mice	double transgenic mouse model of Alzheimer’s disease over expressing amyloid precursor protein, encoding the Swedish mutations at amino acids 595/596 and an exon-9-deleted human PS1
Tg2576 mice	transgenic mouse model, which express a 695-aa residue splice form of human amyloid precursor protein modified by the Swedish Familial AD double mutation K670N-M671L
3xTg-AD mice	triple-transgenic mouse model harboring PS1M146V, APPSwe, and tauP301L transgenes
ATP	adenosine triphosphate
MAPT	microtubule associated protein tau
ROS	reactive oxygen species
IDE	insulin-degrading enzymes
APP	amyloid precursor protein
TZDs	thiazolidinediones
GLP1	glucagon-like peptide-1
DPP-4	dipeptidyl peptidase-4
IGF-1	insulin-like growth factor-1
PS1	presenilin 1
PS 2	presenilin 2
APOE	Apolipoprotein E
PPARs	peroxisome proliferator-activated receptors
AChE	acetylcholinesterase
AMPK	adenosine 5′mmonophosphate-activated protein kinase
PPARs	peroxisome proliferator-activated receptors
PI3K	phosphatidylinositol-3-OH kinase
IRS-2	insulin receptor-2
NLRP3	nod-like receptor pyrin domain containing 3
